# Defining left ventricular remodeling following acute ST-segment elevation myocardial infarction using cardiovascular magnetic resonance

**DOI:** 10.1186/s12968-017-0343-9

**Published:** 2017-03-13

**Authors:** Heerajnarain Bulluck, Yun Yun Go, Gabriele Crimi, Andrew J. Ludman, Stefania Rosmini, Amna Abdel-Gadir, Anish N. Bhuva, Thomas A. Treibel, Marianna Fontana, Silvia Pica, Claudia Raineri, Alex Sirker, Anna S. Herrey, Charlotte Manisty, Ashley Groves, James C. Moon, Derek J. Hausenloy

**Affiliations:** 1 The Hatter Cardiovascular Institute, Institute of Cardiovascular Science, University College London, London, UK; 2 The National Institute of Health Research, University College London Hospitals, Biomedical Research Centre, London, UK; 3 Barts Heart Centre, St Bartholomew’s Hospital, London, UK; 4 Royal Free Hospital, London, UK; 5 Royal Devon and Exeter Hospital, NHS Foundation Trust, Exeter, UK; 6 Struttura Complessa Cardiologia, Fondazione Istituto Di Ricovero e Cura a Carattere Scientifico (IRCCS), Policlinico San Matteo, Pavia, Italy; 7 Multimodality Cardiac Imaging Section, IRCCS Policlinico San Donato, Milan, Italy; 8 UCL Institute of Nuclear Medicine, University College London Hospital, London, UK; 9 National Heart Research Institute Singapore, National Heart Centre Singapore, Singapore, Singapore; 10 Cardiovascular and Metabolic Disorders Program, Duke-National University of Singapore, Singapore, Singapore; 11 Yong Loo Lin School of Medicine, National University Singapore, Singapore, Singapore

**Keywords:** ST-segment elevation myocardial infarction, LV remodeling, trabeculae and papillary muscles, LV end-diastolic volume, LV end-systolic volume, LV ejection fraction, infarct size, microvascular obstruction

## Abstract

**Background:**

The assessment of post-myocardial infarction (MI) left ventricular (LV) remodeling by cardiovascular magnetic resonance (CMR) currently uses criteria defined by echocardiography. Our aim was to provide CMR criteria for assessing LV remodeling following acute MI.

**Methods:**

Firstly, 40 reperfused ST-segment elevation myocardial infarction (STEMI) patients with paired acute (4 ± 2 days) and follow-up (5 ± 2 months) CMR scans were analyzed by 2 independent reviewers and the minimal detectable changes (MDCs) for percentage change in LV end-diastolic volume (%ΔLVEDV), LV end-systolic volume (%ΔLVESV), and LV ejection fraction (%ΔLVEF) between the acute and follow-up scans were determined. Secondly, in 146 reperfused STEMI patients, receiver operator characteristic curve analyses for predicting LVEF <50% at follow-up (as a surrogate for clinical poor clinical outcome) were undertaken to obtain cut-off values for %ΔLVEDV and %ΔLVESV.

**Results:**

The MDCs for %ΔLVEDV, %ΔLVESV, and %ΔLVEF were similar at 12%, 12%, 13%, respectively. The cut-off values for predicting LVEF < 50% at follow-up were 11% for %ΔLVEDV on receiver operating characteristic curve analysis (area under the curve (AUC) 0.75, 95% CI 0.6 to 0.83, sensitivity 72% specificity 70%), and 5% for %ΔLVESV (AUC 0.83, 95% CI 0.77 to 0.90, sensitivity and specificity 78%). Using cut-off MDC values (higher than the clinically important cut-off values) of 12% for both %ΔLVEDV and %ΔLVESV, 4 main patterns of LV remodeling were identified in our cohort: reverse LV remodeling (LVEF predominantly improved); no LV remodeling (LVEF predominantly unchanged); adverse LV remodeling with compensation (LVEF predominantly improved); and adverse LV remodeling (LVEF unchanged or worsened).

**Conclusions:**

The MDCs for %ΔLVEDV and %ΔLVESV between the acute and follow-up CMR scans of 12% each may be used to define adverse or reverse LV remodeling post-STEMI. The MDC for %ΔLVEF of 13%, relative to baseline, provides the minimal effect size required for investigating treatments aimed at improving LVEF following acute STEMI.

**Electronic supplementary material:**

The online version of this article (doi:10.1186/s12968-017-0343-9) contains supplementary material, which is available to authorized users.

## Background

Despite prompt reperfusion of acute ST-elevation myocardial infarction (STEMI) by primary percutaneous coronary intervention (PPCI), adverse left ventricular (LV) remodeling still occurs in a significant proportion of patients [1], and its presence predisposes to heart failure [2] and worse clinical outcomes [3]. In contrast, some reperfused STEMI patients develop reverse LV remodeling, which portends to good clinical outcomes [4].

Cardiovascular magnetic resonance (CMR) is considered the gold-standard imaging modality for quantifying myocardial infarct (MI) size [5], and measuring LV volumes and LV ejection fraction (LVEF) [5, 6], given its high reproducibility [5, 7]. As a result, CMR is increasingly being used to assess surrogate clinical end-points following STEMI in cardioprotection studies. Adverse LV remodeling following STEMI has been conventionally defined as ≥ 20% increase in LV end-diastolic volume (LVEDV) from baseline. This cut-off value was determined using echocardiography, and was based on the upper limit of the 95% confidence interval of intra-observer variability for the percentage change (%Δ) in LVEDV following STEMI [8, 9]. Reverse LV remodeling has been defined as ≥10% decrease in LV end-systolic volume (LVESV) by echocardiography following cardiac resynchronization therapy, and was derived using receiver operator characteristic (ROC) curves for the optimal cut-off for the %ΔLVESV to predict mortality [10]. So far, no cut-off values for adverse and reverse LV remodeling following STEMI have been defined by CMR, and studies using CMR to assess post-STEMI LV remodeling have relied upon using these cut-off values defined by echocardiography for adverse [11, 12] and reverse LV remodeling [13].

Therefore, the first aim of this study was to perform intra-observer and inter-observer measurements of LV parameters in paired acute and follow-up CMR scans in reperfused STEMI patients, in order to determine the minimal detectable changes (MDCs) that could be used as cut-off values for defining post-STEMI remodeling. Secondly, we aimed to identify the cut-off values for clinically important %ΔLVEDV and %ΔLVESV to predict LVEF <50% at follow-up [14], as a surrogate for poor clinical outcome [15]. Finally, cut-off values for %ΔLVEDV and %ΔLVESV were then applied to a large cohort of STEMI patients with paired acute and follow-up scans to assess different patterns of post-STEMI LV remodeling.

## Methods

Patients included in this study have been reported previously in 4 separate studies [16–19] as summarized in the Additional file 1: Online appendix Table 1. All patients provided informed consent at the time of recruitment and the studies were conducted according to the Declaration of Helsinki. Only patients with a paired acute CMR within the first week post PPCI and a follow-up CMR were included in this study.

### Cohort for inter-observer and intra-observer analysis

**Fig. 1:**
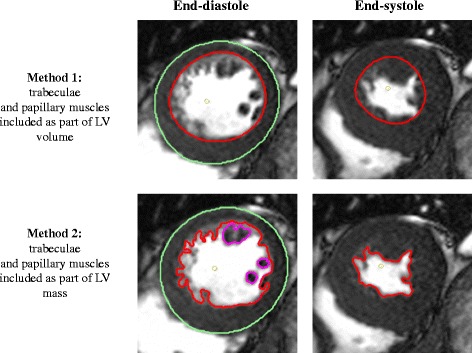
Quantification of LV parameters with T&P part of LV volume (method 1) and part of LV mass (method 2)

Analysis was performed using CVI42 software (Version 5.2.2, Calgary, Canada). Forty STEMI patients reperfused by PPCI, with paired acute and follow-up scans from one of the cohorts reported recently [19–21] were used for inter and intra-observer variability. Semi-automated contours were drawn on the short-axis cine images using the threshold segmentation option for the epicardial border and the automatic detection option for the endocardial border, with manual adjustment when required. The LVEDV, LVESV, LV mass (LVM) and LVEF were quantified using 2 methods as shown in Fig. 1. In method 1, we used rounded endocardial contours and excluded the trabeculae and papillary muscles (T&P) as part of the LVM and they were included as part of the LV volume. In method 2, the T&P were included as part of the LVM and they were excluded from the LV volume. The basal cine slice was included if at least 50% of the cavity circumference was surrounded by ventricular myocardium and this principle was used for both end-systole and end-diastole. %ΔLVEDV, %ΔLVESV, %ΔLVM and %ΔLVEF were calculated as the difference between the follow-up parameters and the corresponding baseline parameters and expressed as a percentage of the baseline parameters. All 40 acute and matching follow-up scans were analysed by 2 experienced CMR operators (twice by HB, 3 years’ experience in CMR, at least 2 months apart and blinded to previous results, and once by YYG, 1 and a half years’ experience in CMR).

### Cohort for LV remodeling

Patient level data were obtained from our 2 previously reported cohorts [18, 19], and from 2 previous randomized controlled trials [16, 17] as listed in the Additional file 1: Online appendix Table 1. Only patients with paired acute and follow-up CMR scans were included. The LV parameters reported by the original studies were used for analysis. All cines were acquired using steady-state free precession-based cines as previously described in their respective publications [16–19]. The CMR details for the acute MI size and microvascular obstruction (MVO) assessment by the 4 different cohorts of patients included are summarized in the Additional file 1: Online appendix Table 1.

### Statistical analysis

Statistical analysis was performed using SPSS version 22 (IBM Corporation, Illinois, US). Normality was assessed using Shapiro-Wilk Test. Continuous data was expressed as mean ± standard deviation (SD) or median (interquartile range) and categorical data was reported as frequencies and percentages. Groups were compared using paired Student *t* test/Wilcoxon signed rank test or unpaired Student *t* test/Mann Whitney *U* test where appropriate. One-way analysis of variance was used to obtain the mean squared error for each LV parameter for inter and intra-observer measurements and their corresponding square root provided their standard error of the measurement (SEM). The 95% confidence interval (CI) for each SEM was calculated as previously described [22]. Coefficient of variation (CoV) was expressed as the standard deviation of the difference divided by the mean and expressed as a percentage and Levene’s test for homogeneity of variance was used to compare CoV between the two methods used for LV parameters quantification (T&P being part of the LV mass or LV volume). Bland-Altman analysis was performed for inter and intra-observer measurements of the LV parameters for comparison. The MDCs with 95% confidence (MDC95) for intra and inter-observer measurements for %ΔLVEDV, %ΔLVESV, % LVM and %ΔLVEF was calculated as 1.96 x SEM x square root of 2. ROC curve analysis was performed to predict an LVEF of <50% at follow-up to identify clinically significant cut-off values for %ΔLVEDV and %ΔLVESV. All statistical tests were two-tailed, and *P* < 0.05 was considered statistically significant.

## Results

The mean age of the 40 STEMI patients used for intra-observer and inter-observer measurements was 59 ± 13 years old and 35 (88%) were male. Details of the paired acute and follow-up CMR scans are shown in Table 1. The acute CMR scan was performed at 4 ± 2 days post-PPCI and the follow-up CMR scan was performed at 5 ± 2 months.

**Table 1:** Characteristics of STEMI patients included for intra-observer and inter-observer study

Details	Number
Number of patients	40
Male (%)	35 (88%)
Age (age)	59 ± 13
Diabetes Mellitus	8 (20%)
Hypertension	14 (35%)
Smoking	12 (30%)
Dyslipidemia	14 (35%)
Chest pain onset to PPCI time (minutes)	267 [122–330]
Infarct artery (%)	
LAD	24 (60%)
RCA	14 (35%)
Cx	2 (5%)
Pre-PPCI TIMI flow (%)	
0	33 (83%)
1	0 (0%)
2	3 (8%)
3	4 (10%)
Post-PPCI TIMI flow (%)	
0	1 (3%)
1	0 (0%)
2	8 (20%)
3	31 (77%)
Acute CMR	
LVEDV/ml	172 ± 38
LVESV/ml	90 ± 30
LVM/g	112 ± 35
LVEF/%	49 ± 8
MVO	26 (65%)
MI size/%LV	27.4 ± 14.6
Follow-up CMR	
LVEDV/ml	182 ± 49
LVESV/ml	88 ± 38
LVM/g	108 ± 26
LVEF/%	53 ± 10
MI size/%LV	19.5 ± 10.5

*PPCI* primary percutaneous coronary intervention, *LAD* left anterior descending artery, *RCA* right coronary artery, *Cx*, circumflex artery, *TIMI*, thrombolysis in myocardial infarction, *CMR* cardiovascular magnetic resonance, *LVEDV*, left ventricular end-diastolic volume, *LVESV* left ventricular end-systole volume, *LVM*, left ventricular mass, *LVEF*, left ventricular ejection fraction, MVO, microvascular obstruction, *MI*, myocardial infarct, *%LV*, percentage of the left ventricle

### Variability of LV parameters between the acute and follow-up CMR scans

Table 2 summarizes the SEM (95%CI), CoV and Bland-Altman analysis of the LV parameters divided into acute and follow-up scans and quantification method. Comparison of CoV did not show any statistical difference for inter-observer or intra-observer measurements (LVEDV, LVESV, LVM, LVEF) on both the acute or follow-up scans between both LV quantification methods (T&P included as part of the LV volume or LV mass) (*P* values between 0.15 and 0.97).

**Table 2:** Intra-observer and inter-observer variability for LV parameters

	Intra- observer			Inter-observer		
	SEM (95% CI)	CoV	Bias ± limits of agreement	SEM (95% CI)	CoV	Bias ± limits of agreement
T&P included as part of the LV volume						
LVEDV						
Acute (*n* = 40)	5.0 (4.1 to 6.4) ml	2.1%	0 ± 7.2 m	5.5 (4.5 to 7.0) ml	2.7%	−2.9 ± 9.6 ml
Chronic (*n* = 40)	5.7 (4.7 to 7.4) ml	2.3%	0.1 ± 8.4 ml	6.3 (5.2 to 8.1) ml	3.3%	0.6 ± 11.8 ml
LVESV						
Acute (*n* = 40)	4.2 (3.5 to 5.4) ml	3.4%	−1.4 ± 6.0 ml	4.5 (3.7 to5.7) ml	4.9%	−0.6 ± 8.8 ml
Chronic (*n* = 40)	5.1 (4.2 to 6.5) ml	3.4%	−0.1 ± 6.0 ml	5.9 (4.8 to 7.6) ml	5.0%	0 ± 8.8 ml
LVM						
Acute (*n* = 40)	7.4 (6.1 to 9.5) g	3.8%	0.3 ± 8.6 g	7.7 (6.3 to 9.9) g	4.4%	−2.2 ± 10.0 g
Chronic (*n* = 40)	6.6 (5.4 to 8.5) g	4.5%	1.4 ± 9.8 g	7.6 (6.1 to 9.8) g	5.0%	−1.8 ± 10.6 g
LVEF						
Acute (*n* = 40)	3.2 (2.7 to 4.2) %	4.1%	0.9 ± 4.0%	2.2 (1.8 to 2.9) %	4.9%	−0.4 ± 4.8%
Chronic (*n* = 40)	1.9 (1.5 to2.4) %	3.2%	0 ± 3.4%	2.7 (2.2 to3.5) %	4.6%	0.2 ± 4.8%
T&P included as part of the LV mass						
LVEDV						
Acute (*n* = 40)	5.1 (4.2 to 6.5) ml	2.6%	−0.4 ± 8.2 ml	5.3 (4.3 to 6.8) ml	3.1%	−2.6 ± 10.0 ml
Chronic (*n* = 40)	4.1 (3.4 to 5.3) ml	2.5%	0.9 ± 8.2 ml	5.2 (4.3 to 6.7) ml	3.5%	−0.4 ± 11.6 ml
LVESV						
Acute (*n* = 40)	4.7 (3.8 to 6.0) ml	3.1%	−0.1 ± 4.8 ml	5.5 (4.5 to 7.0) ml	6.1%	−2.8 ± 9.8 ml
Chronic (*n* = 40)	4.4 (3.6 to 5.6) ml	3.5%	1.0 ± 5.2 ml	5.3 (4.3 to 6.8) ml	6.2%	−2.2 ± 9.6 ml
LVM						
Acute (*n* = 40)	6.3 (5.2 to 8.1) g	3.2%	0.6 ± 8.2 g	7.4 (6.1 to 9.5) g	4.3%	−3.3 ± 11.2 g
Chronic (*n* = 40)	4.0 (3.3 to 5.2) g	3.4%	−0.9 ± 8.0 g	4.2 (3.5 to 5.4) g	5.0%	−1.3 ± 11.8 g
LVEF						
Acute (*n* = 40)	1.7 (1.4 to 2.2) %	3.5%	−0.2 ± 3.6%	2.4 (2.0 to 3.1) %	6.1%	0.9 ± 6.2%
Chronic (*n* = 40)	2.0 (1.6 to 2.6)%	3.2%	−0.2 ± 3.6%	2.6 (2.2 to 3.4) %	5.1%	1.3 ± 5.8%

*SEM* standard error of the measurement, *CoV* coefficient of variation, *LVEDV* left ventricular end-diastolic volume, *LVESV* left ventricular end-systole volume, *LVM* left ventricular mass, *LVEF* left ventricular ejection fraction, *T&P* trabeculae and papillary muscles

**Fig. 2:**
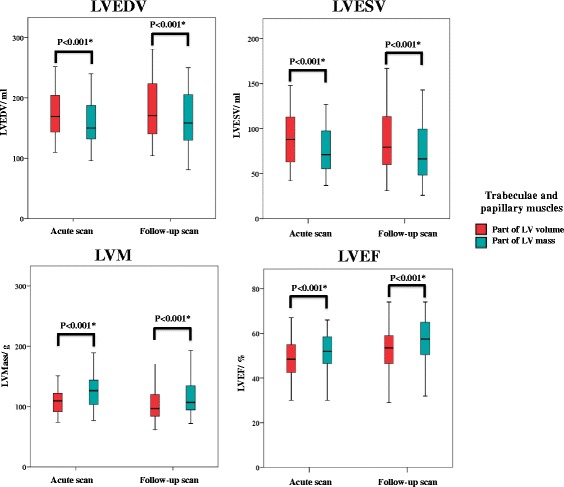
Comparison of LV parameters on the acute and follow-up scans with T&P as part of LV volume or as part of LV mass

The LVEDV and LVESV were significantly higher and the LVM and LVEF were significantly lower both on the acute and follow-up scans when the T&P were included as part of the LV volume as shown in Fig. 2. When they were included as part of the LVM, they contributed the same extent to the LV mass on the acute and the follow-up scans (12.9 ± 5.1 and 11.4 ± 6.3% respectively, *P* = 0.17).

### Variability of %Δ in LV parameters

Details on the intra-observer and inter-observer measurements for %ΔLVEDV, %ΔLVESV, %ΔLVM and %ΔLVEF, both for when T&P was included as part of either LV volume or LV mass, are provided in Table 3. The MDC95 values for these LV parameters were similar for inter- and intra-observer measurements, and whether the T&P were included as part of the LV volume or mass.

**Table 3:** Intra-observer and inter-observer variability for %Δ in LVEDV, LVESV, LVM and LVEF

	Intra- observer			Inter-observer		
	SEM (95%CI)	Bias ± limits of agreement	MDC95	SEM (95%CI)	Bias ± limits of agreement	MDC95
T&P included as part of the LV volume						
%ΔLVEDV (*n* = 40)	3.9 (3.2 to 5.0) %	0.2 ± 6.8%	11%	4.3 (3.6 to 5.6) %	2.2 ± 6.0%	12%
%ΔLVESV (*n* = 40)	3.4 (2.8 to 4.4) %	1.5 ± 8.6%	9%	4.0 (3.3 to 5.1) %	0.7 ± 9.8%	11%
%ΔLVM (*n* = 40)	3.7 (3.1 to 4.8) %	0.9 ± 8.8%	10%	4.1 (3.4 to 5.3) %	0.2 ± 10.6%	11%
%ΔLVEF (*n* = 40)	4.0 (3.2 to 5.1) %	−1.5 ± 9.0%	11%	4.2 (3.5 to 5.4) %	1.8 ± 9.8%	12%
T&P included as part of the LV mass						
%ΔLVEDV (*n* = 40)	3.8 (3.1 to 4.9) %	1.0 ± 8.0%	11%	4.5 (3.7 to 45.7) %	1.5 ± 8.2%	11%
%ΔLVESV (*n* = 40)	3.6 (3.0 to 4.6) %	0.8 ± 8.4%	10%	4.5 (3.7 to 5.8) %	1.1 ± 10.0%	12%
%ΔLVM (*n* = 40)	4.0 (3.3 to 5.2) %	−1.2 ± 9.0%	11%	4.4 (3.6 to 5.6) %	1.3 ± 11.0%	12%
%ΔLVEF (*n* = 40)	4.2 (3.5 to 5.4) %	0 ± 9.8%	12%	4.6 (3.8 to 5.9) %	0.3 ± 9.8%	13%

*SEM* standard error of the measurement, *MDC95* minimal detectable change with 95% confidence, *%Δ* percentage change, *LVEDV* left ventricular end-diastolic volume *LVESV* left ventricular end-systole volume *LVM* left ventricular mass *LVEF* left ventricular ejection fraction *T&P* trabeculae and papillary muscles

Irrespective of how the T&P were dealt with, the highest MDC95 was 11% for %ΔLVEDV and 10% for %ΔLVESV for intra-observer measurements. The corresponding values for inter-observer measurements were 12% for both %ΔLVEDV and %ΔLVESV. Further details for %ΔLVM and %ΔLVEF are provided in Table 4.

**Table 4:** Cut-off values for LVEDV and LVESV in STEMI patients in our cohort (irrespective of whether T&P considered as part of LV volume or LV mass)

	MDC95	
	Intra-observer	Inter-observer
%ΔLVEDV	11%	12%
%ΔLVESV	10%	12%
%ΔLVM	11%	12%
%ΔLVEF	12%	13%

*MDC95* minimal detectable change with 95% confidence, *%Δ* percentage change, *LVEDV* left ventricular end-diastolic volume *LVESV* left ventricular end-systolic volume

### Clinically significant %Δ in LVEDV and LVESV

A total of 146 STEMI patients had matching acute (mean of 4 ± 2 days) and follow-up CMR scans (median of 4 (4–5) months). 12/146 (8%) patients had their scans on a 3 T scanner and the rest were acquired on 1.5 T scanners. Table 5 summarizes the clinical and CMR details of these 146 patients.

**Table 5:** Total number of patients with paired acute and follow-up scan from 4 studies

Details	Number
Number of patients	146
Ludman 2011 [16]	29 (20%)
Crimi 2013 [17]	65 (45%)
Bulluck 2016 [18]	12 (8%)
Bulluck 2016 [19]	40 (27%)
Male	129 (88%)
Age (years)	59 ± 12
Diabetes Mellitus	15 (10%)
Hypertension	67 (46%)
Smoking	64 (44%%)
Dyslipidemia	47 (32%)
Chest pain onset to PPCI time (minutes)	184 [135–282]
Infarct artery (%)	
LAD	109 (75%)
RCA	29 (20%)
Cx	8 (6%)
TIMI flow pre-PPCI	
0	129 (89%)
1	7 (5%)
2	4 (3%)
3	4 (3%)
TIMI flow post-PPCI	
0	2 (1%)
1	2 (1%)
2	23 (16%)
3	117 (82%)
Timing of acute CMR	4 ± 2 days
Timing of follow-up CMR	4 (4–5) months
CMR findings- acute	
LVEDV	156 (132–183) ml
LVESV	80 (64–103) ml
LVM	121 (104–145) g
LVEF	47 ± 9%
MI size	24.6 ± 12.1%LV
MVO	96 (66%)
CMR findings- follow-up	
LVEDV	165 (141–201) ml
LVESV	82 (60–109) ml
LVM	106 (90–132) g
LVEF	50 ± 11%
MI size	17.8 ± 10.1%LV

*PPCI* primary percutaneous coronary intervention, *LAD* left anterior descending artery, *RCA* right coronary artery, *Cx* circumflex artery, *TIMI* thrombolysis in myocardial infarction, *CMR* cardiovascular magnetic resonance, *LVEDV* left ventricular end-diastolic volume *LVESV* left ventricular end-systole volume, *LVM* left ventricular mass, *LVEF* left ventricular ejection fraction, *MI* myocardial infarct, *MVO*, microvascular obstruction

**Fig. 3:**
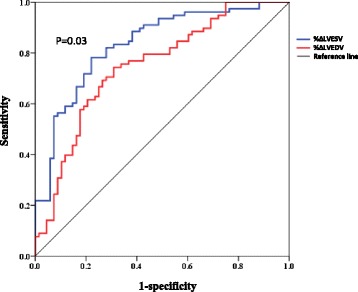
ROC curve comparison for %ΔLVEDV and %ΔLVESV to detect LVEF < 50% at follow-up

ROC curve analysis showed that %ΔLVESV was a better predictor of LVEF of <50% at follow-up, with an area under the curve (AUC) of 0.83 (95% CI 0.77 to 0.90), when compared to an AUC of 0.75 (95% CI 0.67 to 0.83) for %ΔLVESV, *P* = 0.03 for ROC curves comparison (Fig. 3). An 11% increase in %ΔLVEDV had a sensitivity of 72% and a specificity of 70%, and a 5% increase in LVESV had both a sensitivity and specificity of 78%.

These cut-off values were lower than the MDC95 for inter-observer measurements. Therefore, using the cut-off values for MDC95 for inter-observer measurements (given that the scans from different studies were by different observers), an increase in LVEDV of 12% had a sensitivity of 73% and a specificity of 69% and an increase in LVESV of 12% had a sensitivity of 89% and a specificity of 62% to detect an LVEF of <50%.

### Relationship between %ΔLVESV, %ΔLVEDV, %ΔLVEF on post-STEMI LV remodeling

**Fig. 4:**
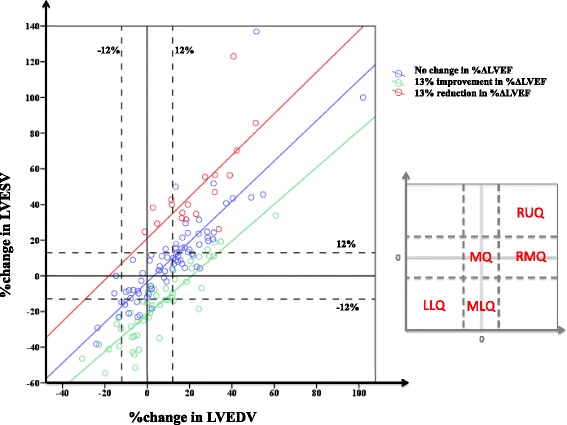
Relationship between %ΔLVEDV, %ΔLVESV and %ΔLVEF. The *vertical dashed lines* represent the cut-off values of +12 and −12% change in LVEDV and the *horizontal dashed lines* represent +12 and −12% %ΔLVESV. Patients were divided into 3 groups for %ΔLVEF based on the MDC95 cut-off of 13%, namely: *blue circles* - no change in LVEF at follow-up; *green circles* - increase in LVEF at follow-up compared to acute scan; *red circles* - decrease in LVEF at follow-up compared to acute scan. Those with a reduction in LVEF at follow-up were more likely to have an increase in both LVEDV and LVESV, and tended to be in the right upper quadrant (RUQ) of the graph. Those with an improvement in LVEF were more likely to have a reduction in LVESV and LVEDV and tended to be in the middle lower quadrant (MLQ) and left lower quadrant (LLQ) of the graph. Some patients had an increase in LVEDV only with or without an improvement in LVEF, and tended to lie in the right middle quadrant (RMQ) of the graph. Those in the middle quadrant (MQ) of the graph had no change in LVEDV or LVESV and predominantly no change in LVEF

Figure 4 shows the relationship between %ΔLVESV and %ΔLVEDV. The dashed lines represent the cut-off values of +12 and −12% change in LVEDV (vertical dashed lines) and +12 and −12% change in LVESV (horizontal dashed lines). Patients were divided into three groups for %ΔLVEF based on the MDC95 cut-off of 13% for inter-observer measurements, namely: blue circles - no change in LVEF at follow-up; green circles - increase in LVEF at follow-up compared to acute scan; red circles - decrease in LVEF at follow-up compared to follow-up. Those with a reduction in LVEF at follow-up were more likely to have an increase in both LVEDV and LVESV, and tended to be in the right upper quadrant (RUQ) of the graph (adverse LV remodeling group). Those with an improvement in LVEF were more likely to have in improvement in LVESV and LVEDV and tended to be in the middle lower quadrant (MLQ) and left lower quadrant (LLQ) of the graph (reverse LV remodeling group). Some patients had an increase in LVEDV only with or without an improvement in LVEF, and tended to lie in the right middle quadrant (RMQ) of the graph (adverse LV remodeling with compensation). Those in the middle quadrant (MQ) of the graph had no change in LVEDV or LVESV and predominantly no change in LVEF (no remodeling group).

**Fig. 5:**
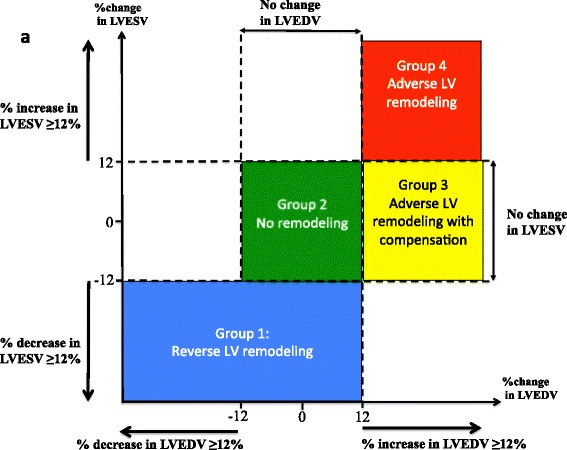
Schematic representation of the different groups of remodeling. Based on the %ΔLVEDV and %ΔLVESV between the follow-up and acute CMR, patients would predominantly fall into these 4 main patterns of LV remodeling groups

**Fig. 6:**
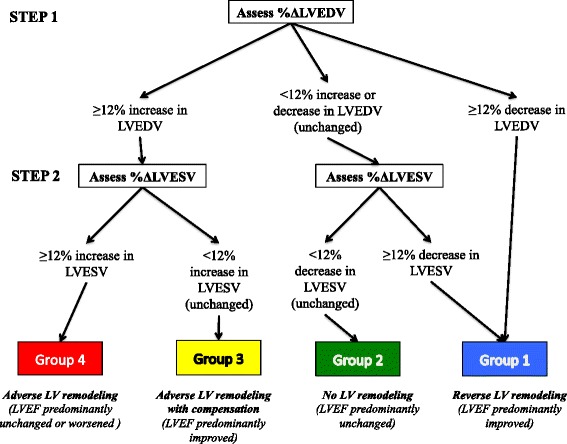
the evaluation of LV remodeling using a 2-step approach. Using a 2-step approach and a combination of %ΔLVEDV and %ΔLVESV, patients can be easily classified into these 4 groups

Figure 5 provides a schematic representation for evaluating LV remodeling post-STEMI from %ΔLVEDV and %ΔLVESV, using a 2-step approach: firstly the %ΔLVEDV is evaluated (using a cut-off value of 12%) and secondly, the %ΔLVESV is assessed as shown in Fig. 6 (using a cut-off value of 12%). Using this approach, 4 main patterns of post-STEMI LV remodeling were observed: Group 1: reverse LV remodeling (with LVEF predominantly improved, 29% of patients); Group 2: no LV remodeling (with LVEF predominantly unchanged, 19% of patients); Group 3: adverse LV remodeling with compensation (with LVEF predominantly improved, 14%); and Group 4: adverse LV remodeling (with LVEF unchanged or worsened, 31%).

### Relationship between %ΔLVESV/%ΔLVEDV, MI size and MVO

**Fig. 7:**
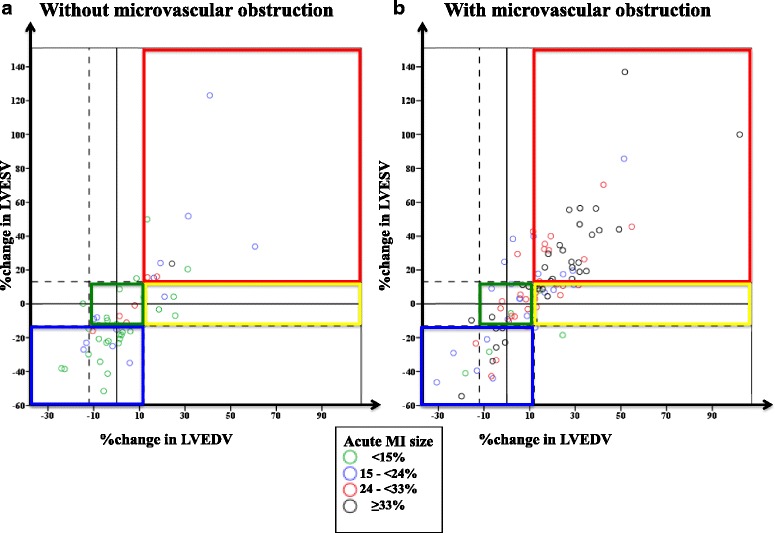
Relation between %ΔLVEDV/%ΔLVEV and different quartiles of acute MI size in (**a**) patients without MVO and (**b**) patients with MVO. Some patients with small MI and no MVO (*green dots* in 6a) developed adverse LV remodeling (falling within the *red box* or *yellow box* in 6a) with others with large MI and MVO (*black dots* in 6b) developed reverse LV remodeling or no remodeling (falling within the *green* or *blue box* in 6b)

The acute MI size was divided into quartiles as follows: <15%, 15 to 24%, 25 to 33% and ≥33%. Figure 7 shows the distribution of acute MI size divided by quartiles and between those without MVO (Fig. 7a) and those with MVO (Fig. 7b). The incidence of MVO on the acute CMR scan was 43%, 66, 81 and 78% for those in Group 1 to 4 respectively, *P* = 0.002. Although those with larger MI size and MVO were more likely to have adverse LV remodeling (Group 4 - red box), there were also patients with small MI sizes and no MVO who went on to develop adverse LV remodeling (green dots within red box in Fig. 7a) and adverse LV remodeling with compensation (Group 3 - within yellow box). Likewise, there were also a notable number of patients with large MI size and MVO who developed reverse LV remodeling (Group 1 - black dots within blue box in Fig. 7b).

## Discussion

The main findings of this study are as follows: (1) The MDC95 in %ΔLVEDV and %ΔLVESV of 12% was higher than the corresponding cut-off values for predicting LVEF < 50% at follow-up (11% for %ΔLVEDV, and 5% for %ΔLVESV), providing cut-off values for assessing adverse and reverse LV remodeling following STEMI by CMR; (2) The MDC95 for %ΔLVM and %ΔLVEF from the acute to follow-up CMR scan were 12% and 13%, respectively, providing cut-off values for assessing changes in these LV parameters following STEMI by CMR; (3) By assessing the combined %ΔLVEDV and %ΔLVESV between the acute and follow-up CMR, we observed 4 different patterns of LV remodeling following STEMI.

In this study, we measured both intra-observer and inter-observer variability, and as expected, the MDC95s for all these LV parameters were greater for inter-observer than intra-observer measurements. Our analyses on the whole cohort mainly focused on the inter-observer rather than the intra-observer measurements because different operators analyzed the scans from each study. We found that the inter-observer MDC95s for %ΔLVEDV and %ΔLVESV between the acute and the follow-up CMR were 12% each. Using these cut-off values for defining LV remodeling following STEMI, a combination of an increase in LVEDV (≥12%) and in LVESV (≥12%) could be used to identify adverse LV remodeling, whereas a decrease in LVESV (≥12%) with or without a decrease in LVEDV (≥12%) could be used to identify reverse LV remodeling. However, further studies are required to investigate the prognostic implications of these proposed cut-off values for defining adverse and reverse LV remodeling following STEMI.

As expected, the cut-off value of 12% or more for %ΔLVEDV to define adverse LV remodeling obtained in our cohort is significantly lower than that defined by echocardiography (20% or more for %ΔLVEDV). This is due to the better spatial resolution of CMR and superior intra-observer and inter-observer variability [6]. On the other hand, the cut-off value for defining reverse LV remodeling as ≥12% for %ΔLVESV from our study is higher than the 10% cut-off value currently proposed by echocardiography [10]. The echocardiography-based method was derived using ROC curve for the optimal cut-off for decrease in ESV to predict mortality in patients undergoing cardiac resynchronization therapy and they did not perform inter-observer and intra-observer variability for change in LVESV. It is highly likely that the inter-observer and intra-observer for %ΔLVESV by echocardiography in STEMI patients would be higher than the CMR cut-off value we obtained.

Currently there is no consensus on whether T&P should be included as part of the LV volume or as part of LV mass during LVEF and LVM assessment by CMR [5]. We therefore provided MDC95 for %ΔLVEDV, %ΔLVESV, %ΔLVM, and %ΔLVEF using both approaches. It is already known that the T&P can significantly affect LV volumes, LV mass, and LVEF [23, 24]. We found that LVEDV and LVESV were higher, and LVM and LVEF were lower when T&P were included as part of the LV volume, and this is consistent with previous reports [23–25]. As their inclusion as part of the LV mass is not always practical depending on the software, both methods are currently considered acceptable [5]. Although the LV parameters differed depending on how the T&P were dealt with, there were no difference in the CoVs both for inter or intra-observer measurements for LVEDV, LVESV, LVM and LVEF when T&P were included as part of the LV volume or LV mass. However, the MDC95 for intra-observer and inter-observer measurements for %Δ in LV parameters varied by 1–2% depending on whether the T&P were included as part of the LV volume or LV mass. We therefore provided the highest MDC95 for each LV parameter in Table 4, irrespective how the T&P were dealt with.

In the absence of clinical outcomes, LVEF <50% in patients with scars have previously been shown to be associated with poor clinical outcomes [15]. Using this cut-off for LVEF at follow-up as a surrogate marker, we obtained cut-off values for %ΔLVEDV and %ΔLVESV of 11 and 5%, respectively. These figures were lower than that defined by our MDC95 cut-off values of 12% for both %ΔLVEDV and %ΔLVESV. The MDC and the clinically significant change are independent of each other as they are derived in different ways and in our case, the former turned out to be larger than the latter. Therefore we chose the cut-off values of MDC95 to define LV remodeling in the whole cohort.

Using the combination of %ΔLVEDV and %ΔLVESV from the acute to the follow-up CMR, we observed 4 different patterns of post-STEMI remodeling (Figs. 5 and 6) The actual impact of these 4 different patterns of post-STEMI LV modeling on clinical outcome will need to be determined in future studies. Conventionally, adverse LV remodeling post-STEMI has been defined by %ΔLVEDV. Our data, suggests that assessing both %ΔLVEDV and %ΔLVESV, may provide further insights into different patterns of LV remodeling following STEMI, thereby allowing one to customize heart failure therapy to prevent adverse LV remodeling or promote reverse LV remodeling. Orn et al. [26] described three patterns of LV remodeling based on presence and persistence of MVO by CMR within the first week of an acute STEMI in a serial CMR study of 42 patients. Most LV remodeling occurred by 2 months and continued to at least 1 year. Those with no MVO had a normal pattern of wound healing; those with MVO on day 2 only, they dilated their ventricle but adapted functionally; and the last group were those with persistent MVO at 1 week and they dilated their ventricle without the ability to adapt functionally. These three groups bear some resemblance to the groups of LV remodeling we identified but we did not have serial CMR data on MVO for comparison. Other factors that determine the pattern of LV remodeling post-STEMI also require further study.

Westman et al. [1] recently showed that there was an imperfect link between MI size and adverse LV remodeling (defined as >10 ml/m^2^ increase in indexed LVEDV). Several studies have also shown that MVO was a strong predictor of adverse LV remodeling [27]. Using the definition in our study for adverse LV remodeling, we also showed that there was an imperfect link between acute MI size and adverse LV remodeling as well between MVO and adverse LV remodeling. Some patients with large MI size and MVO developed reverse LV remodeling and some patients with small MI size and no MVO developed adverse LV remodeling. As eluded by Westman et al. [1], the development of adverse LV remodeling is complex and multi-factorial, and more work is warranted in this field.

We found the MDC95 in %ΔLVM between acute and follow-up scans to be ≥12%, suggesting that this would be the minimal change in LVM that is unlikely due to inter-observer measurement errors. However, the interpretation of changes in LVM following STEMI is complicated by the fact that on the acute scan, the presence of myocardial edema also contributes to the changes in LVM acutely and therefore we did not investigate %ΔLVM in post-STEMI LV remodeling. However, it would be interesting to determine the MDC95 for assessing %ΔLVM in patients with LV hypertrophy related to hypertension or aortic valve disease, in order to provide cut-off values which can be used in studies assessing the regression of LV hypertrophy.

Finally, we found the MDC95 for % ΔLVEF to be ≥13% in STEMI patients when using CMR. This finding suggests that only a relative change in LVEF of 13% or more can be reliably detected by CMR as being beyond inter-observer measurement errors. This is equivalent to an absolute change of 6.5% in a patient with an acute LVEF of 50%. This needs to be taken into consideration when planning future studies designed to investigate new treatments for improving LVEF following STEMI.

### Limitations

Inter-observer and intra-observer measurements were performed in only 40 patients (80 scans) but this is significantly larger than the number of patients used in a previous study (*n* = 10) providing the minimal detectable change in LVEF by echocardiography in patients undergoing chemotherapy (10 patients with echocardiography at 2 time-points) [22]. We only used one analysis tool and LV parameters were quantified using the semi-automated method. We did not have matching echocardiography data for comparison. We did not have complete data on the presence of multi-vessel disease or clinical outcomes and our sample size was relatively small. Therefore, we used an LVEF of <50% at follow-up as a surrogate. [15] There was heterogeneity in the performance of CMR for acute MI size and MVO (scanner strength, dosage and type of contrast, timing of LGE for MVO and MI, quantification technique used – Additional file 1: Online appendix Table 1) and our findings need to be confirmed by future studies.

## Conclusions

The MDCs for %ΔLVEDV and %ΔLVESV between the acute and follow-up CMR scans of 12% each may be used to help define adverse and reverse LV remodeling post-STEMI. Combining %ΔLVEDV and %ΔLVESV following STEMI may provide additional insights into the different pattern of LV remodeling, but their prognostic impact needs to be assessed in future studies. Finally, the MDC for %ΔLVEF of 13% relative to baseline provides the minimal effect size that needs to be taken into consideration when investigating treatments aimed at improving LVEF following acute STEMI.

## Additional file

Additional file 1: Online appendix Table 1: CMR acquisition details of the 4 studies included. (DOCX 26 kb)

## Abbreviations

CI: Confidence interval
CMR: Cardiovascular magnetic resonance
CoV: Coefficient of variation
LV: Left ventricular
LVEDV: LV end-diastolic volume
LVEF: Left ventricular ejection fraction
LVESV: LV end-systolic volume
LVM: Left ventricular mass
MDC: Minimal detectable change
MI: Myocardial infarct
MVO: Microvascular obstruction
PPCI: Primary percutaneous coronary intervention
ROC: Receiver operating characteristics
SD: Standard deviation
SEM: Standard error of measurement
STEMI: ST-elevation myocardial infarction
T&P: Trabeculae and papillary muscles
