# Supplementary material for: Defining left ventricular remodeling following acute ST-segment elevation myocardial infarction using cardiovascular magnetic resonance
Source: J Cardiovasc Magn Reson. 2017 Mar 13;19:26. doi: 10.1186/s12968-017-0343-9 (PMC5346848; doi:10.1186/s12968-017-0343-9)
Supplement: Additional file 1: — Online appendix Table 1: CMR acquisition details of the 4 studies included. (DOCX 26 kb) [file 12968_2017_343_MOESM1_ESM.docx]

**Online appendix table 1:** **CMR acquisition details of the 4 studies included**

|  | **Number of patients with paired CMR** | **Study type** | **Aim** | **Scanner** | **Contrast** | **MVO definition** | **MI size quantification** | **Outcome** |
| --- | --- | --- | --- | --- | --- | --- | --- | --- |
| **Ludman 2011[1]** | 29 patients, 4 months apart | RCT | To assess benefit of erythropoietin during PPCI | 1.5T  Siemens | Dotarem  0.2mmol/kg at 10 minutes | Hypo-intense core on contrast-enhanced images at 10 minutes | Manual | High dose of erythropoietin as an adjunct to PPCI failed to reduce MI size |
| **Crimi 2013[2]** | 65 patients, 4 months apart | RCT | To assess benefit of remote ischemic post conditioning during PPCI | 1.5T  Siemens | Magnevist  0.2mmol/kg at 15 minutes | Hypo-intense core on contrast-enhanced images at 5 minutes | Manual | Remote ischemic conditioning at the time of PPCI was associated with a reduction in enzymatic MI size but this study was underpowered for MI size by CMR |
| **Bulluck 2016[3]** | 12 patients, 1 year apart | Cohort | To assess the role of the remote myocardium with automated extracellular maps and adverse LV remodeling | 3T  Siemens | Dotarem  0.1mmol/kg at 10-15 minutes | Hypo-intense core on contrast-enhanced images at 10-15 minutes | Otsu | 18F-flurodeoxyglucose was reduced in both the reversibly and irreversibly injured myocardium acutely and was reduced in the infarct tissue on the follow-up scan |
| **Bulluck 2016[4]** | 40 patients, 5 months apart | Cohort | To quantify the area at risk using hybrid PET/MR imaging | 1.5T  Siemens | Dotarem  0.1mmol/kg at 10-15 minutes | Hypo-intense core on contrast-enhanced images at 10-15 minutes | 5-SD | ECV in the remote myocardium was increased acutely in STEMI but remained persistently elevated only in those who developed adverse LV remodeling |

RCT: randomized controlled trial; LGE: late gadolinium enhancement; MVO: microvascular obstruction; MI: myocardial infarction; PPCI: primary percutaneous coronary intervention; PET/MR: positron emission tomography/ magnetic resonance; SD: standard deviation

**References**

1. Ludman AJ, Yellon DM, Hasleton J, Ariti C, Babu GG, Boston-Griffiths E, Venugopal V, Walker M, Holdright D, Swanton H, et al: **Effect of erythropoietin as an adjunct to primary percutaneous coronary intervention: a randomised controlled clinical trial.** *Heart* 2011, **97:**1560-1565.

2. Crimi G, Pica S, Raineri C, Bramucci E, De Ferrari GM, Klersy C, Ferlini M, Marinoni B, Repetto A, Romeo M, et al: **Remote ischemic post-conditioning of the lower limb during primary percutaneous coronary intervention safely reduces enzymatic infarct size in anterior myocardial infarction: a randomized controlled trial.** *JACC Cardiovasc Interv* 2013, **6:**1055-1063.

3. Bulluck H, White SK, Frohlich GM, Casson SG, O'Meara C, Newton A, Nicholas J, Weale P, Wan SM, Sirker A, et al: **Quantifying the Area at Risk in Reperfused ST-Segment-Elevation Myocardial Infarction Patients Using Hybrid Cardiac Positron Emission Tomography-Magnetic Resonance Imaging.** *Circ Cardiovasc Imaging* 2016, **9:**e003900.

4. Bulluck H, Rosmini S, Abdel-Gadir A, White SK, Bhuva AN, Treibel TA, Fontana M, Gonzalez-Lopez E, Reant P, Ramlall M, et al: **Automated Extracellular Volume Fraction Mapping Provides Insights Into the Pathophysiology of Left Ventricular Remodeling Post-Reperfused ST-Elevation Myocardial Infarction.** *J Am Heart Assoc* 2016, **5**.
